# A target cultivar-specific identification system based on the chromatographic printed array strip method for eight prominent Japanese citrus cultivars

**DOI:** 10.1270/jsbbs.22065

**Published:** 2023-04-27

**Authors:** Mitsutoshi Okamoto, Yuki Monden, Akiko Shindo, Tomoyuki Takeuchi, Tomoko Endo, Yukinori Shigematsu, Kazuto Takasaki, Hiroshi Fujii, Takehiko Shimada

**Affiliations:** 1 Ehime Research Institute of Citrus Fruits, Yoshida, Uwajima, Ehime 799-3742, Japan; 2 Graduate School of Environmental and Life Science, Okayama University, 1-1-1 Tsushimanaka Kitaku, Okayama, Okayama 700-8530, Japan; 3 FASMAC Co., Ltd., 3088 Okada, Atsugi, Kanagawa 243-0021, Japan; 4 National Agriculture and Food Research Organization Institute of Fruit and Tea Tree Science, Shimizu, Shizuoka 424-0292, Japan

**Keywords:** breeders’ rights, C-Pas, InDel, inspection, next-generation sequencing, protection, retrotransposon

## Abstract

Citrus is a major cultivated crop in Japan, and new cultivars are of great interest in the Japanese and global market. Recently, the infringement of breeders’ rights to citrus cultivars bred in Japan has become a problem related to the agricultural product export strategy promoted by the Japanese government. Cultivar identification systems using DNA markers are an effective tool for protecting breeders’ rights. Here, a novel target cultivar-specific identification system using the chromatographic printed array strip method was developed for eight prominent Japanese citrus cultivars. A polymorphic InDel fragment specific to each cultivar was explored through the screening of published citrus InDel markers and next-generation sequencing of retrotransposon libraries. The cultivar-specific DNA marker set for each cultivar comprised 1–3 polymorphic InDel fragments in combination with a PCR-positive DNA marker for the ribulose-1,5-bisphosphate carboxylase/oxygenase large subunit gene. The DNA markers were detected within 3 hours from DNA extraction to the detection by the C-PAS4 membrane stick following multiplex PCR. The developed system is superior as a convenient, rapid, and cost-effective DNA diagnostic method during inspection. The proposed target cultivar-specific identification system is expected to serve as an efficient tool for the injunction of suspicious registered cultivars, contributing to the protection of breeders’ rights.

## Introduction

Citrus is a member of the family *Rutaceae* with 1,900 species across 160 genera (Groppo *et al.* 2008) and one of the major cultivated crops worldwide. In Japan, the agricultural production of citrus fruits was approximately 201 billion yen, ranking third among Japanese agricultural products, excluding livestock products (Ministry of Agriculture, Forestry and Fisheries 2020). The Japanese citrus industry has been sustained by and has benefited from new cultivars developed by conventional breeding methods. Various new cultivars have been developed in response to the current demands of consumers and producers, such as reduced pest and disease damage in the face of future climate change and premium fruit quality. For instance, ‘Asumi’, ‘Mihaya’, ‘Rinoka’, ‘Asuki’, ‘Ehimekashidai28go’, ‘Kanpei’, ‘Himekoharu’, and ‘Ehimekashidai48go’ have been newly released by the National Agriculture and Food Research Organization Institute (NARO) and Ehime Prefecture, and these cultivars possess improved agricultural traits, such as a higher sugar content, citrus canker resistance, and dripless traits suitable for cut fruit, among others. Such new cultivars have garnered much attraction in both Japanese and global markets. Recently, however, the infringement of breeders’ rights to fruit tree cultivars bred in Japan has become a problem related to the agricultural product export strategy promoted by the Japanese government. As representative cases, the sweet cherry cultivar ‘Benishuho’ and the grape cultivar ‘Shine Muscat’ were illegally transferred and cultivated overseas (Ishikawa 2018, Tahira 2008). Unauthorized overseas outflow of nursery trees leads to subsequent reverse import of pirated fruits into Japan and loss of overseas markets, resulting in potential damage to Japanese farmers. In response to these issues, the Ministry of Agriculture, Forestry and Fisheries has revised a part of the Seedling Law in December 2020 (Ministry of Agriculture, Forestry and Fisheries 2021). The amendment law prohibits the self-propagation of registered cultivars without the permission of the right holder and mandates labeling with the seedling certificate stamp for the prohibition of oversea transfer and restriction on cultivation areas. These restrictions on the handling of registered cultivars (overseas transfer and cultivation) were enacted in April 2021, and propagation based on the permission of registered cultivars came into effect in April 2022.

To safeguard new cultivars more effectively, measures to protect breeders’ rights are imperative. In this context, cultivar identification systems using DNA markers, which can prove the infringement of breeders’ rights, have been developed for various crops. In citrus, various cultivar identification systems have been developed depending on the purpose of use. For instance, in citrus, a cultivar identification system using cleaved amplified polymorphic site (CAPS) markers has been developed (Fujii *et al.* 2019) to inspect nursery trees. Moreover, the National Agriculture and Food Research Organization Institute of the Center for Seeds and Seedlings (NCSS) has published a manual for citrus cultivar identification based on these reports (https://www.naro.go.jp/publicity_report/publication/pamphlet/tech-pamph/130601.html). CAPS markers listed in the manual are guaranteed to be stable and reproducible for citrus cultivar identification to effectively protect breeders’ rights during legal procedures. However, some CAPS markers with long PCR amplicons in the manual cannot be applied to processed products, because PCR amplification is unstable with insufficient quantities of DNA and residual impurities in the DNA extracts. Unless the extracted DNA sample is rendered incompatible because of degradation due to excess heat or chemical treatment, the developed systems are promising for application to both fresh and processed fruit. Additionally, an alternate cultivar identification system using TaqMan-MGB single-nucleotide polymorphism (SNP) genotyping assays has been developed to protect breeders’ rights to the sale of their fruits and processed products overseas (Endo *et al.* 2020). Although these cultivar identification systems contribute to the protection of Japanese citrus cultivar brands, there is a high demand for more convenient and rapid systems to discriminate the target registered cultivar from others at inspection sites, such as in customs. To develop a target cultivar-specific identification system with a simple experimental procedure, a unique DNA polymorphism specific to the target cultivar should be explored, and its detection is desirable for the incorporation of loop-mediated isothermal amplification (LAMP) and chromatographic printed array strip (C-Pas) methods (Notomi *et al.* 2000, Tian *et al.* 2014). To develop DNA markers compatible with these detection methods, sequence information of a relatively long insertion/deletion (InDel) polymorphism specific to the target cultivar is essential.

InDels are derived from the insertion of transposable elements [e.g., miniature inverted repeat transposable elements (MITEs), long interspersed elements (LINEs), and long terminal repeat (LTR) retroelements], polymerase slippage in simple-sequence replication, and unequal crossover events (Britten *et al.* 2003). InDels are an abundant source of genetic markers and are widely distributed across animal and plant genomes. InDel markers are more polymorphic than simple sequence repeats (SSRs) in some crops (Liu *et al.* 2013, Wu *et al.* 2014b). Since InDels can be distinguished based on their size, they have become popular as markers in phylogenetic and genetic studies of crops (Moghaddam *et al.* 2014, Wu *et al.* 2014b, Yamaki *et al.* 2013). In Tartary buckwheat (*Fagopyrum tataricum* Gartn.), PCR-based co-dominant InDel markers were developed from whole-genome resequencing data of 26 accessions and applied to discriminate the genetic resources of this crop (Sohn *et al.* 2021). In strawberry (*Fragaria* × *ananassa* L.), cultivar-specific DNA markers were successfully developed for 8 of the 75 cultivars using retrotransposon insertion polymorphism, and their applicability was verified in processed materials, such as jam (Hirata *et al.* 2020). Ollitrault *et al.* (2012) and Fang *et al.* (2018) identified numerous InDels in citrus by comparing the genome sequences of citrus species and developed PCR-based polymorphic InDel markers. Noda *et al.* (2020) developed 119 indel markers through resequencing analysis of short reads in satsuma mandarin (*Citrus unshiu* Marc.) and used these to identify zygotic embryos. These reports are useful for exploring DNA polymorphisms specific to a target cultivar. However, finding a single DNA polymorphism specific to a target cultivar is difficult, because the citrus breeding population in Japan is generated from repeated crosses among a limited number of ancestral varieties and they partially share common genomes inherited from these ancestors.

To this end, in the present study, we developed cultivar-specific DNA markers based on InDel polymorphism for eight prominent Japanese citrus cultivars (‘Asumi’, ‘Mihaya’, ‘Asuki’, ‘Rinoka’, ‘Ehimekashidai28go’, ‘Kanpei’, ‘Himekoharu’, and ‘Ehimekashidai48go’) and applied these to a rapid detection system using the C-Pas method. In spite of these cultivars have been registered overseas for the protection of breeders’ rights, there are concerns regarding the reimport of illegal fruits. Polymorphic InDel fragments specific to each cultivar were explored through the screening of published citrus InDel markers (Fang *et al.* 2018) and next-generation sequencing (NGS) analysis of libraries of Class I Ty1-copia-type element (CIRE1), Tcs1, and Tcs2 retrotransposon families (Butelli *et al.* 2012, 2017, Horibata and Kato 2020, Rico-Cabanas and Martínez-Izquierdo 2007). The cultivar-specific DNA marker set for each cultivar comprised 1–3 polymorphic InDel fragments in combination with a PCR-positive DNA marker for the ribulose-1,5-bisphosphate carboxylase/oxygenase large subunit (*rbcL*) gene in the chloroplast genome. DNA markers were detected within 10 min on the C-PAS4 membrane stick after multiplex PCR. This simple and rapid detection system using the C-Pas method can be helpful to assess infringement at inspection sites regardless of whether the test sample is a target cultivar. The proposed target cultivar-specific identification system will reinforce the protection of recently released prominent Japanese citrus cultivars.

## Materials and Methods

### Plant material and DNA extraction

A total of 26 citrus cultivars (Table 1) preserved at the Division of Citrus Research of the NARO Institute of Fruit and Tea Tree Science (NIFTS) and Ehime Prefecture were used in the present study. Taxonomic classification followed Tanaka’s system (Tanaka 1969). Sample accession numbers (JP no.) and registration numbers (registration no.) in Table 1 are based on GeneBank (https://www.gene.affrc.go.jp/databases-plant_search.php) at the Genetic Resources Center, National Agriculture and Food Research Organization, and Plant Variety Protection Office (http://www.hinshu2.maff.go.jp/en/en_top.html) of the Ministry of Agriculture, Forestry and Fisheries of Japan (MAFF). Genomic DNA was isolated from fully expanded fruit and peel tissues using the DNeasy Plant Mini Kit (Qiagen, Hilden, Germany).

### Screening of cultivar-specific InDel polymorphisms in published markers

A total of 185 InDel markers published in previous research (Fang *et al.* 2018, Kawahara *et al.* 2020) were applied for the screening of polymorphic InDel fragments specific to the target cultivar using genomic DNA of 26 citrus cultivars (Table 1). A single polymorphic InDel fragment or a minimum combination of polymorphic InDel fragments was screened to identify the target cultivar among the 26 cultivars. The PCR mixture was prepared in at a final volume of 10 μL, containing 5 ng of genomic DNA, 5 pmol of the forward and reverse primers, and 5 μL of KOD One PCR Master Mix (TOYOBO, Osaka, Japan). The cycling conditions were as follows: 32 cycles of denaturation at 98°C for 10 s, annealing at 56°C for 5 s, and extension at 68°C for 2 s and one cycle of final extension at 68°C for 30 s. All reactions were performed on the ProFLEX PCR system (Thermo Fisher Scientific, Waltham, MA, USA). The PCR products were electrophoresed on 2.0% agarose gel (Agarose standard 01, Solana; Rikaken, Nagoya, Japan) and visualized through ethidium bromide staining.

### Construction of a sequencing library for retrotransposon insertion sites

To comprehensively determine retrotransposon insertion sites, the flanking regions of insertion sites were amplified using PCR, and the amplified products were sequenced on the Illumina HiSeq platform. A sequencing library was constructed according to previously described methods (Monden *et al.* 2014, 2015). In the present study, three retrotransposon families (CIRE1, Tcs1, and Tcs2) were targeted for sequencing and a sequencing library was prepared for each retrotransposon family. First, genomic DNA was fragmented using NEBNext dsDNA Fragmentase (1,000 bp–2,000 bp) (New England Biolabs, Inc., MA, USA), and forked adaptors were ligated to the fragmented DNA. The forked adaptors were prepared by annealing two different oligos (Forked_Type1 and Forked_Com) (Supplemental Table 1). Primary PCR amplification was performed with retrotransposon-specific (CIRE1-PPT or Tcs-PBS) and adaptor-specific (AP2-Type1) primer combinations and adaptor-ligated DNA as the template (Supplemental Table 1). For primary PCR amplification, a common primer (Tcs-PBS) was used to prepare the sequencing library for the Tcs1 and Tcs2 families. Nested PCR amplification was performed using tailed PCR primers (P5 and P7 primers) and primary PCR products as the template. The tailed PCR primers contained the P5 or P7 sequence (Illumina) for hybridization on the sequencing flow cell, Rd1SP or Rd2SP sequence (Illumina) for sequencing primer-binding sites, and several barcodes for multiplex sequencing. Thus, retrotransposon-specific primers (i.e., D501–D508) comprised a P5 sequence, barcode sequence, Rd1SP sequence, and retrotransposon end sequence, while the adapter-specific primers (i.e., D701–D713) comprised a P7 sequence, barcode sequence, Rd2SP sequence, and adapter sequence. The primer combinations used for each sample are listed in Supplemental Table 2. The PCR products were size-selected (500–1,000 bp) on agarose gels and purified using the QIAquick Gel Extraction Kit (QIAGEN). The purified products were then quantified using the Qubit fluorometer (Invitrogen, Carlsbad, CA, USA), and the size selection range was confirmed using the Agilent 2200 TapeStation system (Agilent, Santa Clara, CA, USA). A HiSeq sequencing library was prepared by pooling equal amounts of purified barcoded products from each line. HiSeq reads for analyzing retrotransposon insertion sites were deposited under accession number DDBJ: DRA014490 (CIRE1), DRA014491 (Tcs1), and DRA014492 (Tcs2).

### Data analysis for retrotransposon insertion polymorphisms

The resulting paired-end reads (150 bp) were analyzed following procedures described in previous studies (Hirata *et al.* 2020, Monden *et al.* 2014, 2015, Sasai *et al.* 2019). The obtained reads were analyzed using Maser—a pipeline execution system in the Cell Innovation Program of the National Institute of Genetics (https://cell-innovation.nig.ac.jp/index_en.html). Adaptor trimming and quality filtering (QV ≥30) were performed using Cutadapt (Martin 2011) and FLEXBAR (Dodt *et al.* 2012), respectively. The filtered reads were trimmed to a specific length (50 bp) covering most of the sequences. Reads with 10 or more identical sequences were reduced to a single sequence in FASTA format and then clustered using the BLAT self-alignment program (Kent 2002) under the following parameter settings: “-tileSize” = 8, “-minMatch” = 1, “-minScore” = 10, “-repMatch” = –1, and “-oneOff” = 2. This clustering analysis produced several clusters, each corresponding to a specific retrotransposon insertion site. Next, an optimal threshold was set to evaluate the presence or absence of retrotransposon insertions; if the number of reads in a given cluster at a specific insertion site accounted for <0.1% of all reads for that cultivar, then retrotransposons were considered to be absent from that site. This yielded genotyping information for the presence (1) *versus* absence (0) of retrotransposon insertions in all cultivars. Finally, cultivar-specific and polymorphic insertion sites among the cultivars were screened based on this genotyping information.

### Development and PCR validation of cultivar-specific DNA markers

Polymorphic InDel fragments were cloned into a T-vector (pMD19, Takara, Tokyo, Japan) and subjected to Sanger sequencing. The plasmid was prepared using the QIAprep Spin Miniprep kit (Qiagen), and sequencing was performed using the ABI PRISM 3130xl Genetic Analyzer (Applied Biosystems, Foster City, CA, USA) according to the manufacturer’s instructions. The obtained sequences were blasted against the Mikan genome database (https://mikan.dna.affrc.go.jp/), and the allelic sequences of polymorphic InDel fragments that had been inserted or deleted were collected. Following sequence alignment, the junction site of insertion or deletion was determined for each polymorphic InDel fragment, and a polymorphic fragment-specific primer set was designed using the sequence around this junction site and non-homologous sequence between the allelic sequences. For retrotransposon DNA polymorphism, forward and reverse primers were designed based on the sequence of either the 5ʹ-LTR region of the retrotransposon or cultivar-specific retrotransposon insertion sites. A PCR-positive DNA marker was designed using the coding region of the *rbcL* gene in the citrus chloroplast genome. Primer sequences of the developed DNA markers are provided in Table 2. The developed cultivar-specific DNA markers were validated using PCR according to the above conditions, followed by 2.0% agarose gel electrophoresis.

### Multiplex PCR and detection of DNA markers using the C-Pas method

To detect PCR products using the C-Pas method, single-stranded tag and spacer sequences were added to the 5ʹ-end of either the forward or the reverse primer for each DNA marker. Specific DNA markers for each target cultivar were amplified with PCR-positive DNA markers using multiplex PCR. The primer concentration ratio and multiplex PCR conditions were optimized for uniform amplification of DNA markers. PCR was performed according to the manufacturer’s protocol using a premixture primer supplied in the kit (FASMAC, Kanagawa, Japan). PCR products with the tag–spacer sequence for single-stranded tag hybridization were mixed with the dye and developing solution (Tohoku Bio-Array, Miyagi, Japan). Subsequently, a C-PAS4 membrane stick (Tohoku Bio-Array) was dipped into the developing solution for 10 min.

## Results

### Screening of cultivar-specific DNA markers for eight prominent Japanese citrus cultivars

Of the 185 InDel markers in previous reports (Fang *et al.* 2018, Kawahara *et al.* 2020), polymorphic InDel fragments specific to the target cultivars were screened using 26 widely distributed citrus cultivars in the Japanese market, which account for over 94% of all citrus fruit shipments produced in the country (Table 1). Most InDel markers revealed 2–4 multiple PCR fragment patterns. Since the Japanese breeding population partially shares the genomes inherited from limited ancestral varieties, a single polymorphic InDel fragment specific to the target cultivar was expected to be rarely detected unless their parents were rarely used as seed or pollen parents in conventional Japanese breeding, such as lemon and grapefruit. A single polymorphic InDel fragment was obtained in ‘Rinoka’, which is generated from the cross between lemon and hyuganatsu (*C. tamurana* hort. ex Tanaka). The IND141 marker generated three InDel fragments among 26 cultivars, and only ‘Rinoka’ possessed a 600 bp fragment (IND141-l). There was no single polymorphic InDel fragment in the remaining target seven cultivars.

### NGS of transposon libraries and screening of cultivar-specific sequence polymorphisms induced through the insertion of retrotransposons in eight prominent Japanese citrus cultivars

Since finding a single polymorphic InDel fragment to identify the target cultivar was difficult in the screening of published InDel markers, the LTR retrotransposon families CIRE1, Tcs1, and Tcs2 were selected for comprehensive analysis of insertion sites using a high-throughput sequencing platform. In the CIRE1 library, 122,668,732 reads of 150 bp were obtained (min = 2,597,900, average = 4,718,028, and max = 6,110,395 reads per line) (Supplemental Table 3). After preprocessing, 57,420,094 reads remained (Supplemental Table 4). These reads were used for clustering analysis using the BLAT self-alignment program (Kent 2002), producing 93 independent insertion sites in 26 cultivars (Supplemental Table 4). Among these insertion sites, the following 10 were considered putative cultivar-specific insertions: four insertions (CIRE1-CL30, CIRE1-CL28, CIRE1-CL21, and CIRE1-CL55) specific to ‘Haruhi’; two insertions (CIRE1-CL15 and CIRE1-P51) specific to ‘Kawanonatsudaidai’; two insertions (CIRE1-P60 and CIRE1-P75) specific to ‘Rinoka’; and one insertion each specific to ‘Reikou’ (CIRE1-CL48) and grapefruit (CIRE1-P58). In the Tcs1 library, 75,646,143 (min = 1,529,440, average = 2,909,467, and max = 5,003,387 reads per line) reads of 150 bp were obtained (Supplemental Table 3). After preprocessing, 37,259,503 reads remained (Supplemental Table 5). These reads were used for clustering analysis with the BLAT self-alignment program (Kent 2002), producing 276 independent insertion sites of Tcs1 in 26 cultivars (Supplemental Table 5). Among these, the following 24 insertion sites were considered to be cultivar-specific: eight insertions (Tcs1-P104, Tcs1-CL32, Tcs1-P110, Tcs1-P112, Tcs1-CL46, Tcs1-CL64, Tcs1-P148, and Tcs1-P151) specific to lemon; three insertions (Tcs1-CL57, Tcs1-CL68, and Tcs1-CL93) specific to ‘Haruhi’; two insertions (Tcs1-CL85 and Tcs1-P95) specific to ‘Kawanonatsudaidai’; two insertions (Tcs1-CL87 and Tcs1-CL69) specific to ‘Rinoka’; two insertions (Tcs1-CL113 and Tcs1-P99) specific to ‘Ehimekashidai28go’; one insertion (Tcs1-CL90) specific to grapefruit; one insertion (Tcs1-P107) specific to ‘Shiranuhi’; one insertion (Tcs1-CL83) specific to ‘Iyo’; one insertion (Tcs1-CL119) specific to ‘Hassaku’; one insertion (Tcs1-P118) specific to ‘Ponkan’; one insertion (Tcs1-CL59) specific to ‘Reikou’; and one insertion (Tcs1-CL58) specific to ‘Himekoharu’. In the Tcs2 library, 134,858,060 (min = 3,671,674, average = 5,186,848, max = 6,797,994 reads per line) reads of 150 bp were obtained (Supplemental Table 3). After preprocessing, 33,251,578 reads remained (Supplemental Table 6). Clustering analysis using these reads produced 174 independent insertion sites for Tcs2 in 26 cultivars (Supplemental Table 6). Among these, the following 19 insertion sites were considered to be cultivar-specific: four insertions (Tcs2-CL74, Tcs2-CL88, Tcs2-CL81, and Tcs2-CL99) specific to lemon; three insertions (Tcs2-CL52, Tcs2-CL70, and Tcs2-CL77) specific to grapefruit; three insertions (Tcs2-CL79, Tcs2-CL85, and Tcs2-P97) specific to ‘Rinoka’; three insertions (Tcs2-CL18, Tcs2-CL41, and Tcs2-P100) specific to ‘Haruhi’; two insertions (Tcs2-CL82 and Tcs2-CL105) specific to ‘Kawanonatsudaidai’; two insertions (Tcs2-CL73 and Tcs2-CL68) specific to ‘Ehimekashidai28go’; one insertion (Tcs2-CL83) specific to ‘Reikou’; and one insertion (Tcs2-CL80) specific to ‘Himekoharu’. Overall, the three LTR retrotransposon families exhibited high sequence polymorphisms induced by the insertion of retrotransposons among the 26 citrus cultivars, and a number of cultivar-specific sequence polymorphisms were detected among the three families.

Furthermore, candidate cultivar-specific insertion polymorphisms were obtained through the NGS analysis of the three retrotransposon libraries. The following candidate cultivar-specific sequence polymorphisms were obtained in three cultivars: seven insertion sites in ‘Rinoka’; four insertion sites in ‘Ehimekashidai28go’; and two insertion sites in ‘Himekoharu’. The Tcs1 and Tcs2 retrotransposon libraries tended to be more polymorphic than the CIRE1 library among the 26 cultivars tested.

### Cultivar-specific DNA marker sets combined with multiple polymorphisms in the remaining five cultivars

Obtaining cultivar-specific DNA polymorphisms comprising a single PCR fragment was difficult in the remaining five cultivars owing to their similar genetic backgrounds, which was attributed to repeated crossbreeding among selected mandarin germplasms. To acquire cultivar-specific DNA marker sets in the remaining five cultivars, DNA polymorphism data of InDel fragments and retrotransposon insertion sites were analyzed using the MinimalMarker program (Fujii *et al.* 2013). Two or three markers were necessary to identify the target among the 26 cultivars. For instance, two polymorphic InDel fragments [a 350 bp fragment of Cp0419 (Cp0419-s) and a 250 bp fragment of IND214 (IND214-s)] were the minimum set required to discriminate ‘Asumi’ among the 26 cultivars. Notably, ‘Asumi’ alone possessed these two polymorphic InDel fragments among the 26 cultivars, and the presence or absence of these two polymorphic InDel fragments may allow identifying whether the examined sample is ‘Asumi’. ‘Mihaya’ may be identified based on the presence of two polymorphic insertion sites (Tcs1-P86 and Tcs2-CL55-M). ‘Asuki’ may be identified based on the presence of a polymorphic insertion site (Tcs2-CL25) and a 300 bp PCR fragment of IND235 (IND235-s). ‘Ehimekashidai48go’ may be identified by the presence of two polymorphic insertion sites (Tcs1-CL26 and Tcs2-CL55-E). Only ‘Kanpei’ required the following three DNA polymorphisms for its identification among the 26 cultivars: a 350 bp PCR fragment of Cp0419 (Cp0419-s), a 280 bp PCR fragment of IND265 (IND265-l), and a 160 bp PCR fragment of IND44 (IND44-l).

### Development of cultivar-specific DNA markers and PCR validation

**Fig. 1.**
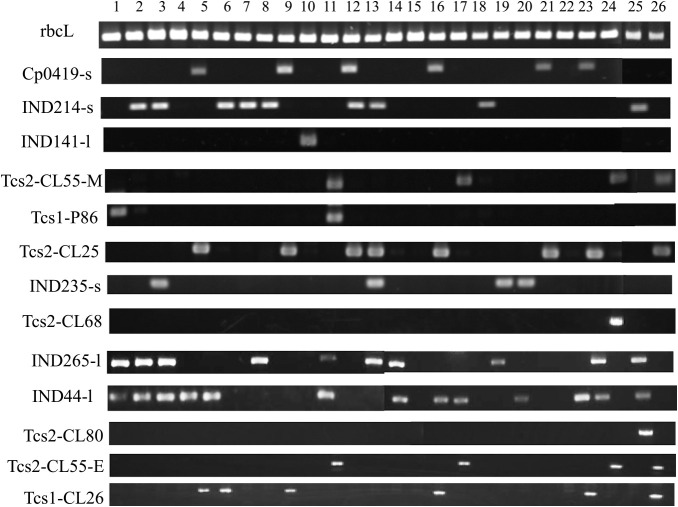
PCR validation of cultivar-specific DNA polymorphisms. PCR products were electrophoresis on 2% agarose gel and visualized using ethidium bromide staining. The number indicates plant material in Table 1.

The cultivar-specific DNA polymorphisms listed in Table 2 were finally selected, and their PCR primers were designed for compatible detection using 2% agarose gel electrophoresis and C-PAS4 membrane stick. The PCR fragment size of each DNA marker was adjusted such that they did not overlap in 2% agarose gel electrophoresis. The position of each DNA marker in the clementine mandarin genome sequence (Wu *et al.* 2014a) was determined through blast search analysis against the Mikan Genome Database. PCR validation was performed to confirm whether the obtained cultivar-specific DNA markers can work for each cultivar in actual PCR. The PCR validation test was performed to determine whether only the target polymorphic PCR fragment can be amplified among the 26 cultivars. The PCR products were electrophoresed on 2% agarose gel (Fig. 1). In ‘Rinoka’, ‘Ehimekashidai28go’, and ‘Himekoharu’, the primer sets for IND141-l, Tcs2-CL68, and Tcs2-CL80 were confirmed to amplify a single polymorphic PCR fragment specific to each cultivar. For the remaining five cultivars, primer design was successful in amplifying target polymorphic fragments specifically, and the PCR products were detected in the expected cultivars, consistent with the results of DNA polymorphism screening described above. The presence or absence of PCR products for these DNA markers in the 26 cultivars is summarized in Table 3.

### Multiplex PCR and C-Pas detection of cultivar-specific DNA marker sets for eight prominent Japanese citrus cultivars

**Fig. 2.**
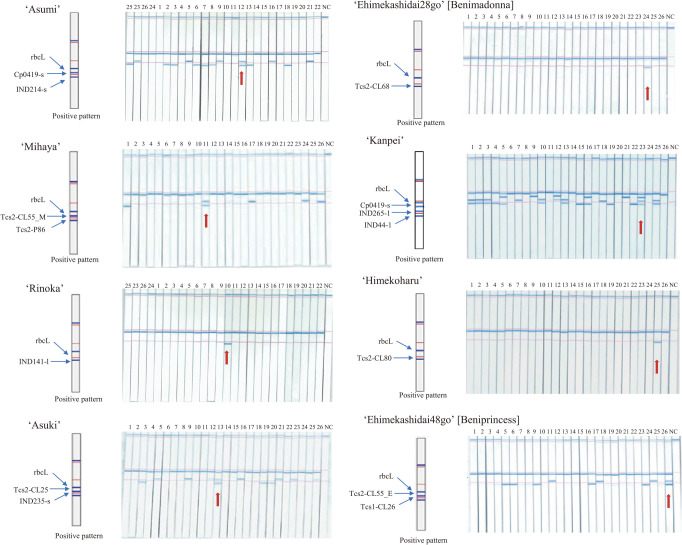
Detection pattern of the C-Pas detection kit for eight prominent Japanese citrus cultivars. The number indicates plant material in Table 1. Genomic DNA isolated from leaves was used. NC indicates non-template DNA. Red arrow indicates the target cultivar in each C-Pas detection kit.

Eight cultivar-specific DNA marker sets to identify the target among the 26 citrus cultivars were developed from a single DNA polymorphism or combined multiple DNA polymorphisms. Since DNA diagnostics is based on the presence or absence of polymorphic PCR fragments, the *rbcL* gene in the chloroplast genome was utilized as a PCR-positive DNA marker to determine whether the multiplex PCR experiment itself is running appropriately. The primer concentration ratio was optimized for each cultivar-specific DNA marker set to realize uniform amplification of multiple PCR fragments. The PCR products were detected using C-PAS4 membrane stick. Specifically, 1 μL of PCR solution was added to 20 μL of developing solution in a 1.5 mL tube and mixed by pipetting. Subsequently, the C-PAS4 membrane stick was placed in the solution for 10 min. The signal was visualized as blue bands. For ‘Rinoka’, ‘Ehimekashidai28go’, and ‘Himekoharu’, a single cultivar-specific PCR fragment and positive DNA marker were simultaneously amplified in multiplex PCR. In C-Pas detection, two bands were detected on the C-PAS4 membrane stick for these cultivars, whereas only a single band was detected for the other cultivars (Fig. 2). For ‘Asumi’, ‘Mihaya’, ‘Asuki’, and ‘Ehimekashidai48go’, two cultivar-specific PCR fragments and positive DNA marker were simultaneously amplified in multiplex PCR. Three bands were detected on the C-PAS4 membrane stick for these cultivars, whereas only one or two bands were detected for the other cultivars. For ‘Kanpei’, three cultivar-specific PCR fragments and positive DNA marker were simultaneously amplified in multiplex PCR. Four bands were detected on the C-PAS4 membrane stick for this cultivar, whereas one to three bands were detected for the other cultivars. Therefore, DNA polymorphisms of all makers were consistent with results in Table 3 and patterns in 2% agarose gel electrophoresis (Supplemental Fig. 1).

### C-PAS detection of cultivar-specific DNA marker sets using genomic DNA isolated from citrus peel

**Fig. 3.**
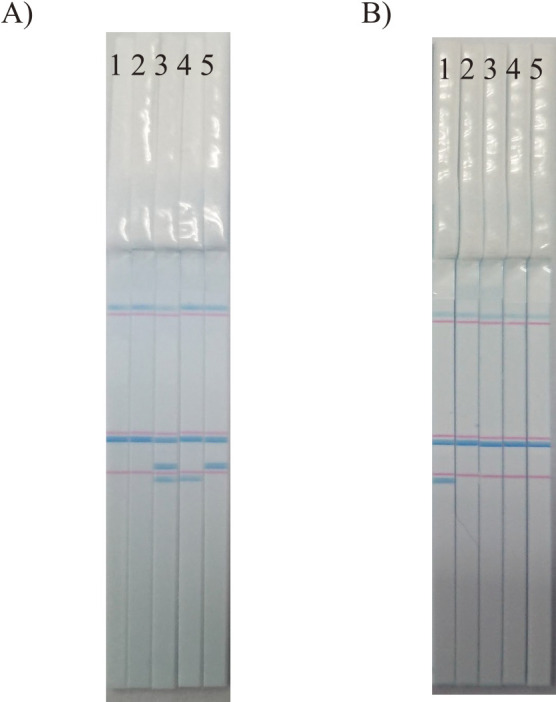
Representative detection pattern of the C-Pas detection kit for ‘Asumi’ (A) and ‘Rinoka’ (B) using genomic DNA isolated from peel tissues. 1: ‘Rinoka’, 2: ‘Mihaya’, 3: ‘Asumi’, 4: ‘Asuki’, and 5: ‘Kanpei’.

The developed C-Pas detection kit for eight target cultivars was applied to DNA samples isolated from fruit tissues to confirm its applicability for the inspection of fruits. Briefly, genomic DNA was isolated from fruit peel tissues using the DNeasy Plant Mini Kit (Qiagen) owing to higher quantity and less residual impurities in the DNA extracts than other fruit tissues. PCR and PCR product detection were performed according to the protocols supplied in each C-Pas detection kit. Fig. 3 shows the representative results for ‘Asumi’ and ‘Rinoka’. The DNA samples of ‘Rinoka’, ‘Mihaya’, ‘Asumi’, ‘Asuki’, and ‘Kanpei’ were used for evaluation. With the C-Pas detection kit for ‘Asumi’, only ‘Asumi’ presented three blue bands corresponding to *rbcL*, Cp0419, and IND214, while the remaining cultivars presented either of these bands and the PCR-positive marker for *rbcL*. With the C-Pas detection kit for ‘Rinoka’, only ‘Rinoka’ presented two blue bands corresponding to *rbcL* and IND141-l, while the remaining cultivars presented a single band corresponding to *rbcL*. Similarly, C-Pas detection kits for the remaining target cultivars were confirmed to present the same detection patterns between the DNA samples isolated from leaf and fruit tissues (data not shown). Thus, these C-Pas detection kits can indeed be used to inspect fruits.

## Discussion

The infringement of breeders’ rights to new cultivars of agricultural crops bred in Japan has been a concern regarding the agricultural product export strategy promoted by the Japanese government. In the case of citrus, the scions of newly registered cultivars are illegally shipped overseas, and the reverse import of pirated fruits and processed products into Japan is a serious problem. We have already developed two cultivar identification systems using CAPS and TaqMan-MGB SNP markers to inspect suspicious nursery trees and fruits in the market (Endo *et al.* 2020, Fujii *et al.* 2019). These systems could precisely identify at the DNA level the suspicious nursery trees or fruits that infringe breeders’ rights. However, these DNA diagnostics requires a certain amount of time to obtain the genotyping information of plural DNA markers for cultivar identification. In this context, a rapid target cultivar-specific identification system must be developed for suspected imported fresh fruits at customs, because the procedure of injunction for perishables, such suspicious fruits of a registered cultivar, must be completed within 3 days of notification from customs. DNA diagnostics using the newly developed system is a very simple experimental procedure involving PCR and C-Pas detection. It can be completed within an hour of DNA extraction and simply answers whether the tested sample is a target cultivar. In this newly developed system, the enzymatic reaction of PCR amplicons, expensive chemical reagents, and special analysis instruments are not required; therefore, it is a more convenient DNA diagnostic system than CAPS- and SNP-based systems. Therefore, the developed system can enable rapid inspection of suspicious imported fruits at customs.

In a target cultivar-specific identification system, it is desirable to find and use a single cultivar-specific DNA polymorphism; however, only three of the examined eight cultivars could be used in the present study. Conventional citrus varieties comprise a complex mosaic genome structure derived from repeated natural crosses among a limited number of ancestors (Curk *et al.* 2014). Japanese citrus cultivars have been developed through repeated crosses among the comparatively narrow germplasms of 13 conventional varieties (Imai *et al.* 2017). Therefore, most Japanese citrus cultivars may partially share certain genomes of these ancestral varieties, rendering the detection of a single cultivar-specific DNA polymorphism difficult. Regarding the three successful cultivars ‘Rinoka’, ‘Ehimekashidai28go’, and ‘Himekoharu’, one of their parent or ancestral parent is lemon, grapefruit, or ‘Ougonkan’ (*C. sp*), respectively, which are rarely used as a breeding parent in Japan. In plants, retrotransposons can be separated into two major subclasses, namely LTR-RTs and non-LTR RTs, based on their structure and transposition mechanisms (Todorovska 2007). Majority of the LTR-RTs can be further divided into Copia and Gypsy superfamilies (Domingues *et al.* 2012). Several types of LTR-RT molecular markers have been developed in citrus, including inter-retrotransposon amplified polymorphism (IRAP), sequence-specific amplified polymorphism (SSAP), and retrotransposon-microsatellite amplified polymorphism (REMAP), among others (Biswas *et al.* 2010, Du *et al.* 2018, Horibata and Kato 2020). In the present study, three retrotransposon families (CIRE1, Tcs1, and Tcs2) were investigated using NGS analysis because they constitute transcriptionally active retrotransposons in citrus. The citrus CIRE1 family has been confirmed as one of the few transcriptionally active retrotransposons reported in plants and presents the typical structural features of Copia retrotransposons, with approximately 5 kbp of LTRs and other intrinsic elements (Rico-Cabanas and Martínez-Izquierdo 2007). There are seven families, namely CIRE1–CIRE7, share over 85% nucleotide identity. CIRE1 exhibits root-specific expression in sweet orange plants, and wounding and exogenous application of plant hormones, such as methyl jasmonate and auxin, has been reported to enhance CIRE1 transcription in leaf tissues. In addition, Tcs1 and Tcs2 have been confirmed to be transcriptionally active retrotransposons in blood oranges (Butelli *et al.* 2012). They possess approximately 5 kbp LTRs with all features of a typical Copia-like retrotransposon. Tcs1 transcripts were detected in the fruit, but not in the leaves, of blood oranges. Elevated Tcs1 transcript levels were observed in ‘Tarocco’ and ‘Moro’ blood orange varieties following cold storage, indicating that Tcs1 and Tcs1-like elements are activated by cold and likely contribute to temperature-dependent anthocyanin accumulation in blood oranges. Considering the transcriptional expression patterns of these three retrotransposons, Tcs1 and Tcs2 may be more activate than CIRE1 under citrus cultivation conditions characterized by low temperatures during autumn and winter in Japan. Similar result is observed in this study that a total of 10 insertions for single cultivar-specific DNA polymorphism to address 4 cultivars were obtained in CIRE1 library among the examined 26 cultivars, while 24 insertions for 12 cultivars in Tcs1 library and 19 insertions for 8 cultivars in Tcs2 library (data not shown).

In the present study, retrotransposon insertion sites were sequenced through short-read sequencing to identify a single cultivar-specific InDel polymorphism. Additionally, various candidate cultivar-specific DNA polymorphisms were obtained *in silico* for citrus cultivars other than the eight target cultivars in the NGS analysis of the three retrotransposon families. Therefore, this method is a powerful tool for identifying cultivar-specific DNA polymorphisms. In particular, almost all retrotransposon DNA markers were identified at approximate positions on clementine genome using in search analysis of their sequence reads. Furthermore, these DNA polymorphisms were successfully converted to DNA markers for detection using the C-Pas method, but could not be applied to the LAMP method owing to insufficient cultivar-specific unique sequence information for the design of LAMP primer sets. LAMP is an isothermal amplification method designed to detect a target nucleic acid without a special analyzing instrument and provides a rapid, sensitive, and specific DNA polymorphism detection (Notomi *et al.* 2000). The design of LAMP primers requires comparatively longer polymorphic sequence specific to the target cultivar owing to refer the six regions in the target sequence. Recent advances in long-read sequencing technologies, such as PacBio and Oxford Nanopore Technologies, allow for assembling long repetitive DNA fragments onto reference genomes. Moreover, using chromosome conformation capture (Hi-C) technology in combination with a genetic map, contigs can be assembled at the chromosome scale. In combination with these new technologies, the precise positions of transposon insertion sites can be identified within long repetitive genetic elements, providing important sequence information for the development of cultivar-specific DNA markers for detection using the LAMP method. In addition, technological improvements for assigning transposons, retrotransposons, and repeat elements to accurate positions in the draft sequences are expected to be pivotal for elucidating the molecular mechanisms of sport mutations in citrus trees and developing cultivar identification systems for sport mutant and nucellar cultivars.

## Author Contribution Statement

Mr. Mitsutoshi Okamoto investigated DNA polymorphisms among four cultivars bred in the Ehime Prefecture to develop cultivar-specific DNA markers for them and is the first author. Dr. Yuki Monden analyzed retrotransposon libraries using NGS to identify cultivar-specific DNA polymorphisms induced by retrotransposon insertion and is the co-first author. Ms. Akiko Shindo constructed retrotransposon libraries and analyzed them using NGS to identify cultivar-specific DNA polymorphisms induced by retrotransposon insertion. Dr. Tomoyuki Takeuchi developed the C-Pas detection kit to adjust the primer concentration ratio in multiplex PCR for four cultivars bred at NARO. Dr. Tomoko Endo investigated DNA polymorphisms among four cultivars bred at NARO and developed cultivar-specific DNA markers for them. Dr. Yukinori Shigematsu investigated the applicability of the C-Pas detection kit to fruit samples from four cultivars bred in the Ehime Prefecture. Dr. Kazuto Takasaki developed the C-Pas detection kit to adjust the primer concentration ratio in multiplex PCR for four cultivars bred in the Ehime Prefecture. Dr. Hiroshi Fujii calculated the cultivar-specific minimum set of DNA markers using MinimalMarker. Dr. Takehiko Shimada investigated the applicability of the C-Pas detection kit to fruit samples from four cultivars bred at NARO and is the corresponding author to organize the manuscript.

## Supplementary Material

Supplemental Figure

Supplemental Tables

## Figures and Tables

**Table 1.** Citrus cultivars used in the experiment

No.	JP No.*^a^*	Registration No.*^b^*	Cultivar name (conventional variety)	Parentage or scientific name*^c^*
1	117351		‘Miyagawa-wase’ (Satsuma mandarin)	*Citrus unshiu* Marc.
2	168864		‘Duncan’ grapefruit (Grapefruit)	*C. paradisi* Macf.
3	172154		‘Trovita’ orange (Sweet orange)	*C. sinensis* (L.) Osbeck
4	117289		‘Lisbon’ lemon (Lemon)	*C. limon* (L.) Burm. f.
5	117159		‘Shiranuhi’ [Dekopon*^d^*]	‘Kiyomi’ × ‘Nakano 3 gou’ ponkan
6	117373		Iyo (Iyo)	*C. iyo* hort. ex Tanaka
7	117297		‘Kawanonatsudaidai’ (Natsudaidai)	*C. natsudaidai* Hayata
8	117286		Hassaku (Hassaku)	*C. hassaku* hort. ex Tanaka
9	171505	413	‘Ohta ponkan’ (Ponkan)	*C. reticulata* Blanco
10		24081	‘Rinoka’	‘Lisbon’ lemon × Hyuganatsu
11	251815	23722	‘Mihaya’	‘Tsunonozomi’ × No. 1408
12	245233	23723	‘Asumi’	Okitsu 46 gou × ‘Harumi’
13		29242	‘Asuki’	Okitsu 46 gou × ‘Harumi’
14		13542	‘Reikou’	Unknown × ‘Murcott’
15		17970	‘Tsunokagayaki’	KyOw No. 14 × ‘Encore’
16		17969	‘Seinannohikari’	EnOw No. 21 × ‘Youkou’
17		20788	‘Tsunonozomi’	‘Kiyomi’ × ‘Encore’
18	237599	20679	‘Haruhi’	Okitsu 46 gou × ‘Awa-orange’
19	115521		‘Kiyomi’	‘Miyagawa-wase’ × ‘Trovita’ orange
20	118842	9398	‘Setoka’	KyEn No. 4 × ‘Murcott’
21	117468	7506	‘Harumi’	‘Kiyomi’ × ponkan F-2432
22		12069	‘Harehime’	E-647 × ‘Miyagawa-wase’
23		15548	‘Kanpei’	‘Nishinokaori’ × Ponkan
24		12981	‘Ehimekashidai28go’ [Benimadonna*^e^*]	‘Nankou’ × ‘Amakusa’
25		17067	‘Himekoharu’	‘Kiyomi’ × ‘Ougonkan’
26		29243	‘Ehimekashidai48go’ [Beniprincess*^f^*]	‘Ehimekashidai28go’ × ‘Kanpei’

*^a^* Discription by Genebank (http://www.gene.affrc.go.jp/databases-plant_search.php). *^b^* Discription by Plant Variety Protection Office at MAFF, Japan (http://www.hinshu2.maff.go.jp/en/en_top.html). *^c^* Tanaka’s system was used for scientific name. *^d^* The registered trademark of the Federation of Kumamoto Prefectural Fruit Agriculture Cooperatives. *^e^* The registered trademark of National Federation of Agricultural Cooperative Associations. *^f^* The registered trademark of Ehime Prefectural Headquarters.

**Table 2.** Information of DNA markers which identify the target cultivar among 26 citrus cultivars

Cultivar name	DNA marker	Forward primer	Reverse primer	Fragment size (bp)	Primer position of clementine genome ver1.0*^a^*	
					Scaffold	Primer position*^b^*
	rbcL	ACAGAGACTAAAGCGAGTGTTG	TAAATGGTTGGGAGTTCACGTTC	622	–	–
‘Asumi’	Cp0419-s	AGCAATGGCAACACGATGTTCCTTTG	CGGTTACCAAATTGTTAACAGCTATC	371	5	28816349::28816374
	IND214-s	AAACTTGGGTTGTGTAAG	TGCGGTTTTGTCTTTCCCTTTTATACG	246	1	106523::106547
‘Rinoka’	IND141-l	GTAGATAGCATTACCAATAAAGTGTT	GAGAGACACCTTTAAGTCTTTTTTGG	246	3	1477572::1477597
‘Mihaya’	Tcs2-CL55_M	TGGATGAAAAATGAGGGTGGGAAGC	TTGAATGGTAAAGTATTTGCACG	279	1	8581874::8581896
	Tcs1-P86	TCAAATGTAGTTGCCAACTTC	GTCCTTATTTCTGGTTTCATGT	182	9	1185498::1185519
‘Asuki’	Tcs2-CL25	ACTCTCAATTCTCTCTACAA	TCTGTATCTTGAAAAGAGATTAGCC	243	3	10838788::10838812
	IND235-s	TGCTGAATCAAGATCACCACAACG	CCTGTTCAAATGTTATTATACATG	144	5	6038219::6038242
‘Ehimekashidai28go’ [Benimadonna*^c^*]	Tcs2-CL68	GGTGGGAAGCCCTCTTTATATAGC	GATGAACCATAACCCTAATACACTACAATG	185	3	30278648::30278677
‘Kanpei’	Cp0419-s	AGCAATGGCAACACGATGTTCCTTTG	CGGTTACCAAATTGTTAACAGCTATC	246	5	28816349::28816374
	IND265-l	ATGCATCACAAATTTGTTT	TGGTTGGCGGCTAATGAATA	280	8	962119::962137
	IND44-l	TGTTAGCCTGCTAGACTTT	TCAATAGATTCACCGTGCATT	152	7	3264327::3264347
‘Himekoharu’	Tcs2-CL80	CTCAACTCCCTAAATTGTTCTCTCA	GACTAATGGTGGCACAATTATATTGATAG	262	8	4106127::4106155
‘Ehimekashidai48go’ [Beniprincess*^d^*]	Tcs2-CL55_E	GTGGGAGCCCTCTTTATATAGC	TTGAATGGTAAAGTATTTGCACG	263	1	8581890::8581896
	Tcs1-CL26	GTTGTTGCCAACTTCTTCTTCC	CCAATATCATGTAGGAATATTTCATTCC	160	1	8581874::8581896

*^a^* Phytozome (https://phytozome.jgi.doe.gov/pz/portal.html) for clementine genome information. *^b^* Primer position on one side which is coserved on clementine genome. *^c^* The registered trademark of National Federation of Agricultural Cooperative Associations. *^d^* The registered trademark of Ehime Prefectural Headquarters. ND: Primer position is not determined due to the multiple hits by blast search analysis on Mikan Genome Database (https://mikan.dna.affrc.go.jp/).

**Table 3.** DNA polymorphisms of DNA markers which identify the target cultivar among 26 citrus cultivars

Cultivar name	DNA marker	DNA polymorphism*^a^*																									
		1	2	3	4	5	6	7	8	9	10	11	12	13	14	15	16	17	18	19	20	21	22	23	24	25	26
	rbcL	○	○	○	○	○	○	○	○	○	○	○	○	○	○	○	○	○	○	○	○	○	○	○	○	○	○
‘Asumi’	Cp0419-s	×	×	×	×	○	×	×	×	○	×	×	○	×	×	×	○	×	×	×	×	○	×	○	×	×	×
	IND214-s	×	○	○	×	×	○	○	○	×	×	×	○	○	×	×	×	×	○	×	×	×	×	×	×	○	×
‘Rinoka’	IND141-l	×	×	×	×	×	×	×	×	×	○	×	×	×	×	×	×	×	×	×	×	×	×	×	×	×	×
‘Mihaya’	Tcs2-CL55_M	×	×	×	×	×	×	×	×	×	×	○	×	×	×	×	×	○	×	×	×	×	×	×	○	×	○
	Tcs1-P86	○	×	×	×	×	×	×	×	×	×	○	×	×	×	×	×	×	×	×	×	×	×	×	×	×	×
‘Asuki’	Tcs2-CL25	×	×	×	×	○	×	×	×	○	×	×	○	○	×	×	○	×	×	×	×	○	×	○	×	×	○
	IND235-s	×	×	○	×	×	×	×	×	×	×	×	×	○	×	×	×	×	×	○	○	×	×	×	×	×	×
‘Ehimekashidai28go’ [Benimadonna*^b^*]	Tcs2-CL68	×	×	×	×	×	×	×	×	×	×	×	×	×	×	×	×	×	×	×	×	×	×	×	○	×	×
‘Kanpei’	Cp0419-s	×	×	×	×	○	×	×	×	○	×	×	○	×	×	×	○	×	×	×	×	○	×	○	×	×	×
	IND265-l	○	○	○	×	×	×	○	×	×	○	×	○	○	×	×	×	×	○	×	×	×	×	○	×	○	×
	IND44-l	○	○	○	○	○	×	×	×	×	○	×	×	○	×	○	○	×	×	○	×	×	○	○	×	○	×
‘Himekoharu’	Tcs2-CL80	×	×	×	×	×	×	×	×	×	×	×	×	×	×	×	×	×	×	×	×	×	×	×	×	○	×
‘Ehimekashidai48go’ [Beniprincess*^c^*]	Tcs2-CL55_E	×	×	×	×	×	×	×	×	×	×	○	×	×	×	×	×	○	×	×	×	×	×	×	○	×	○
	Tcs1-CL26	×	×	×	×	○	○	×	×	○	×	×	×	×	×	×	○	×	×	×	×	×	×	○	×	×	○

*^a^* Number indicates cultivars listed in Table 1. *^b^* The registered trademark of National Federation of Agricultural Cooperative Associations. *^c^* The registered trademark of Ehime Prefectural Headquarters. ○: Presence of PCR fragment, ×: Absense of PCR fragment.
