# Supplementary material for: A target cultivar-specific identification system based on the chromatographic printed array strip method for eight prominent Japanese citrus cultivars
Source: Breed Sci. 2023 Apr 27;73(2):146–57. doi: 10.1270/jsbbs.22065 (PMC10316311; doi:10.1270/jsbbs.22065)
Supplement: Supplementary file 1 — Supplemental Figure [file 73_146_s1.pdf]

doi: 10.1270/jsbbs.22065

“Asumi”

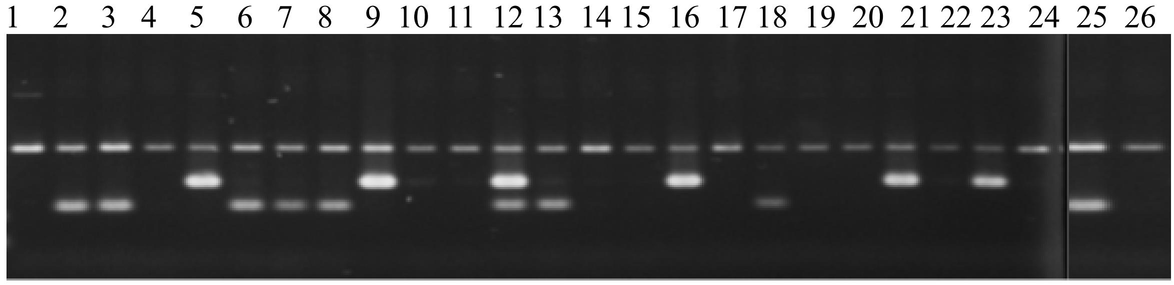

“Mihaya”

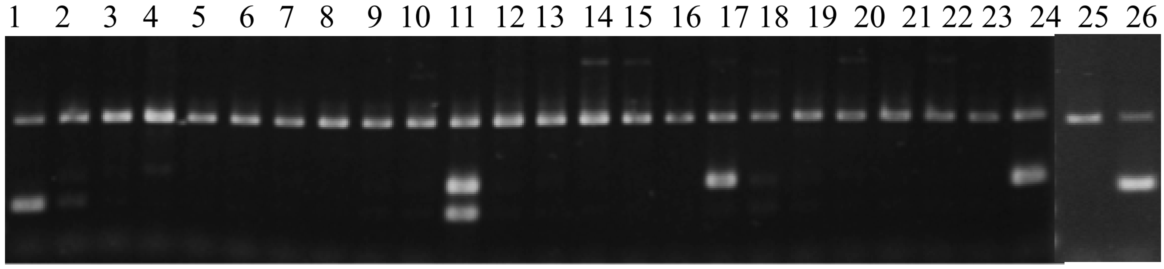

“Rinoka”

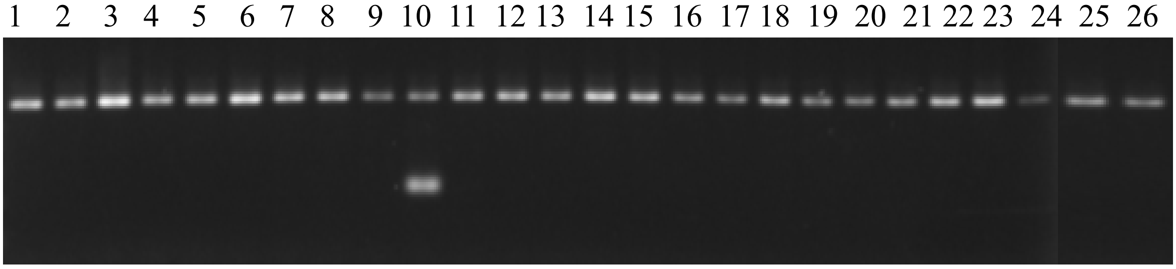

“Asuki”

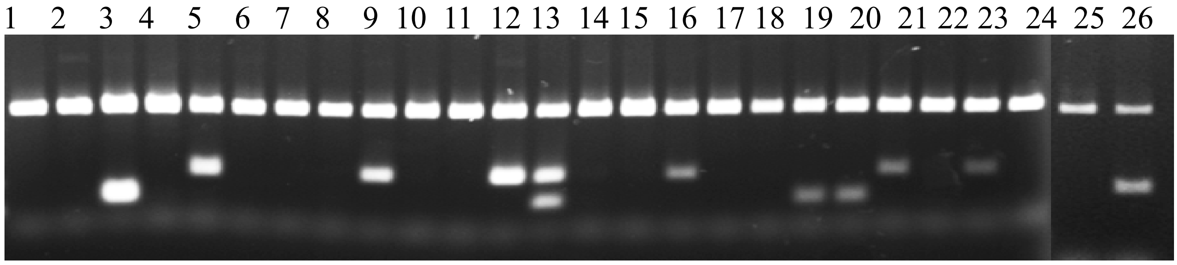

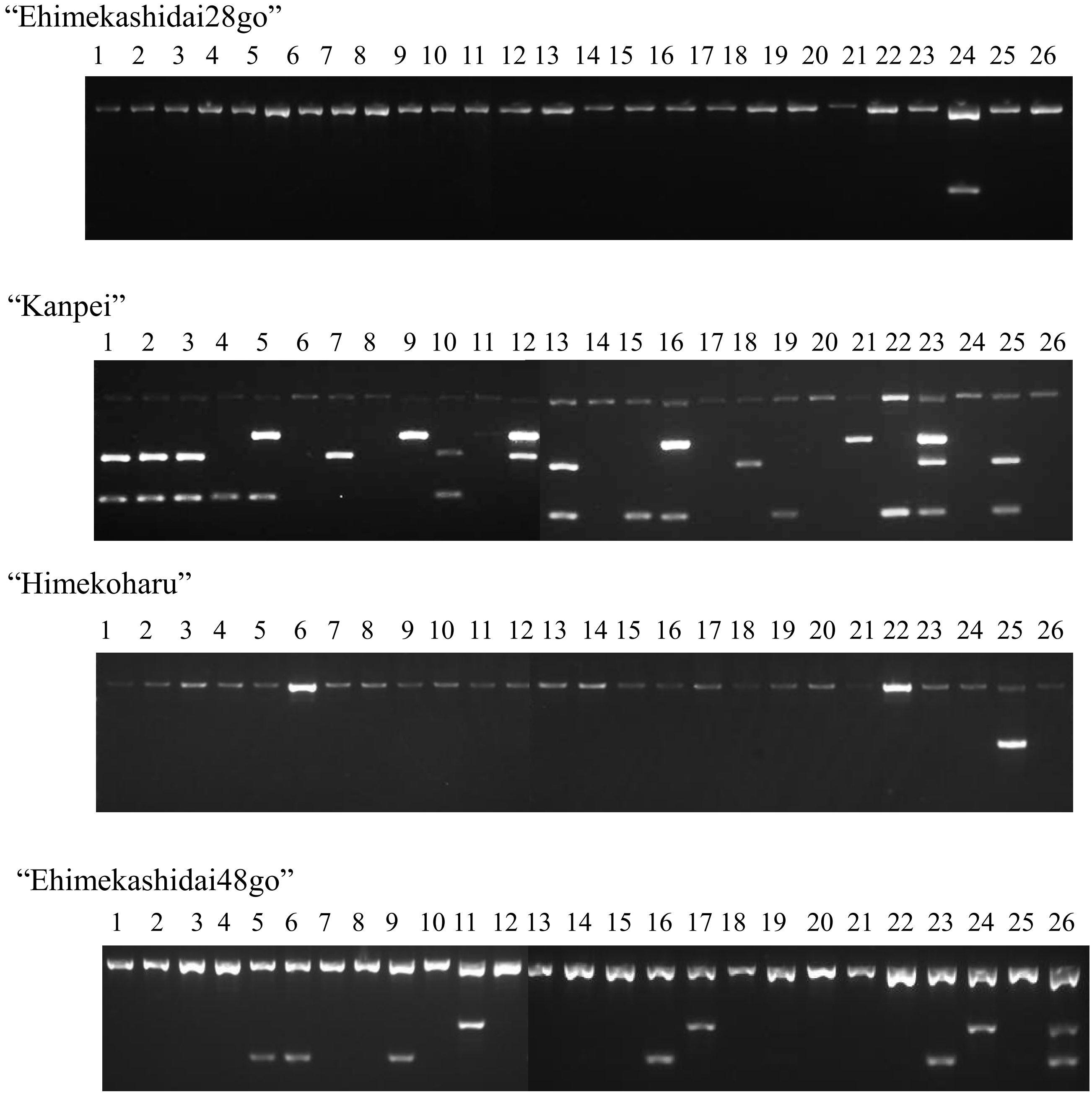

Supplemental Fig. 1. PCR fragment patterns of cultivar-specific DNA markers for eight prominent Japanese citrus cultivars. Multiplex PCR was performed using DNA markers listed in Table 2. PCR products were electrophoresis on 2% agarose gel and visualized using ethidium bromide staining. The number indicates plant material in Table 1.
