# Supplementary material for: A target cultivar-specific identification system based on the chromatographic printed array strip method for eight prominent Japanese citrus cultivars
Source: Breed Sci. 2023 Apr 27;73(2):146–57. doi: 10.1270/jsbbs.22065 (PMC10316311; doi:10.1270/jsbbs.22065)
Supplement: Supplementary file 2 — Supplemental Tables [file 73_146_s2.pdf]

doi: 10.1270/jsbbs.22065

Supplemental Table 1. Primers used for library construction.

Experimental process	Primer name	Sequence	Genbank accession number
Adaptor ligation	Forked_Type1[a]	aatagggctcgagcggcagctattaatagtaCt	
	Forked_Com[b]	GTACTATTAATAGCATCTTCGTTCGTCGAT	
Primary PCR	Tcs-PBS	GCTCTGATACCACTGTTAGGAAATTGG	JN402332.1, JN402333.1
	CIRE1-PPT	CTGCAAGGTGGAGATTGTGAAG	AM040263.2
	AP2-Type1	AATAGGGCTCGAGCGGC	
Nested PCR	D501_R1_Tcs1	AATGATACGGCGACCACCGAGATCTACACTATAGCCTACACTCTTTCCCTACACGACGCTCTTCCGATCTCTTGTGGCCACCGACTTTGACA	JN402332.1
	D502_R1_Tcs1	AATGATACGGCGACCACCGAGATCTACACATAGAGGCACACTCTTTCCCTACACGACGCTCTTCCGATCTCTTGTGGCCACCGACTTTGACA	JN402332.1
	D504_R1_Tcs2	AATGATACGGCGACCACCGAGATCTACACGGCTCTGAACACTCTTTCCCTACACGACGCTCTTCCGATCTGGCGCCAGCCATGACTTTCA	JN402333.1
	D506_R1_Tcs2	AATGATACGGCGACCACCGAGATCTACACTAATCTTAACACTCTTTCCCTACACGACGCTCTTCCGATCTGGCGCCAGCCATGACTTTCA	JN402333.1
	D507_R1_CIRE1	AATGATACGGCGACCACCGAGATCTACACCAGGACGTACACTCTTTCCCTACACGACGCTCTTCCGATCTCGTGAGTCTTGCTGTGGTTTAGACT	AM040263.2
	D508_R1_CIRE1	AATGATACGGCGACCACCGAGATCTACACGTACTGACACACTCTTTCCCTACACGACGCTCTTCCGATCTCGTGAGTCTTGCTGTGGTTTAGACT	AM040263.2
	D701_R2_1	CAAGCAGAAGACGGCATACGAGATATTACTCGGTGACTGGAGTTCAGACGTGTGCTCTTCCGATCTGGGCTCGAGCGGCAGCTATTAATAGTACT	
	D702_R2_1	CAAGCAGAAGACGGCATACGAGATTCCGGAGAGTGACTGGAGTTCAGACGTGTGCTCTTCCGATCTGGGCTCGAGCGGCAGCTATTAATAGTACT	
	D703_R2_1	CAAGCAGAAGACGGCATACGAGATCGCTCATTGTGACTGGAGTTCAGACGTGTGCTCTTCCGATCTGGGCTCGAGCGGCAGCTATTAATAGTACT	
	D704_R2_1	CAAGCAGAAGACGGCATACGAGATGAGATTCCGTGACTGGAGTTCAGACGTGTGCTCTTCCGATCTGGGCTCGAGCGGCAGCTATTAATAGTACT	
	D705_R2_1	CAAGCAGAAGACGGCATACGAGATATTCAGAAGTGACTGGAGTTCAGACGTGTGCTCTTCCGATCTGGGCTCGAGCGGCAGCTATTAATAGTACT	
	D706_R2_1	CAAGCAGAAGACGGCATACGAGATGAATTCGTGTGACTGGAGTTCAGACGTGTGCTCTTCCGATCTGGGCTCGAGCGGCAGCTATTAATAGTACT	
	D707_R2_1	CAAGCAGAAGACGGCATACGAGATCTGAAGCTGTGACTGGAGTTCAGACGTGTGCTCTTCCGATCTGGGCTCGAGCGGCAGCTATTAATAGTACT	
	D708_R2_1	CAAGCAGAAGACGGCATACGAGATTAATGCGCGTGACTGGAGTTCAGACGTGTGCTCTTCCGATCTGGGCTCGAGCGGCAGCTATTAATAGTACT	
	D709_R2_1	CAAGCAGAAGACGGCATACGAGATCGGCTATGGTGACTGGAGTTCAGACGTGTGCTCTTCCGATCTGGGCTCGAGCGGCAGCTATTAATAGTACT	
	D710_R2_1	CAAGCAGAAGACGGCATACGAGATTCCGCGAAGTGACTGGAGTTCAGACGTGTGCTCTTCCGATCTGGGCTCGAGCGGCAGCTATTAATAGTACT	
	D711_R2_1	CAAGCAGAAGACGGCATACGAGATTCTCGCGCGTGACTGGAGTTCAGACGTGTGCTCTTCCGATCTGGGCTCGAGCGGCAGCTATTAATAGTACT	
	D712_R2_1	CAAGCAGAAGACGGCATACGAGATAGCGATAGGTGACTGGAGTTCAGACGTGTGCTCTTCCGATCTGGGCTCGAGCGGCAGCTATTAATAGTACT	
	D713_R2_1	CAAGCAGAAGACGGCATACGAGATGAATAATCGTGACTGGAGTTCAGACGTGTGCTCTTCCGATCTGGGCTCGAGCGGCAGCTATTAATAGTACT	
	D701_R2	CAAGCAGAAGACGGCATACGAGATCGAGTAATGTGACTGGAGTTCAGACGTGTGCTCTTCCGATCTGGGCTCGAGCGGCAGCTATTAATAGTACT	
	D702_R2	CAAGCAGAAGACGGCATACGAGATTCTCCGGAGTGACTGGAGTTCAGACGTGTGCTCTTCCGATCTGGGCTCGAGCGGCAGCTATTAATAGTACT	
	D703_R2	CAAGCAGAAGACGGCATACGAGATAATGAGCGGTGACTGGAGTTCAGACGTGTGCTCTTCCGATCTGGGCTCGAGCGGCAGCTATTAATAGTACT	
	D704_R2	CAAGCAGAAGACGGCATACGAGATGGAATCTCGTGACTGGAGTTCAGACGTGTGCTCTTCCGATCTGGGCTCGAGCGGCAGCTATTAATAGTACT	
	D705_R2	CAAGCAGAAGACGGCATACGAGATTTCTGAATGTGACTGGAGTTCAGACGTGTGCTCTTCCGATCTGGGCTCGAGCGGCAGCTATTAATAGTACT	
	D706_R2	CAAGCAGAAGACGGCATACGAGATACGAATTCGTGACTGGAGTTCAGACGTGTGCTCTTCCGATCTGGGCTCGAGCGGCAGCTATTAATAGTACT	
	D707_R2	CAAGCAGAAGACGGCATACGAGATAGCTTCAGGTGACTGGAGTTCAGACGTGTGCTCTTCCGATCTGGGCTCGAGCGGCAGCTATTAATAGTACT	
	D708_R2	CAAGCAGAAGACGGCATACGAGATGCGCATTAGTGACTGGAGTTCAGACGTGTGCTCTTCCGATCTGGGCTCGAGCGGCAGCTATTAATAGTACT	
	D709_R2	CAAGCAGAAGACGGCATACGAGATCATAGCCGGTGACTGGAGTTCAGACGTGTGCTCTTCCGATCTGGGCTCGAGCGGCAGCTATTAATAGTACT	
	D710_R2	CAAGCAGAAGACGGCATACGAGATTTCGCGGAGTGACTGGAGTTCAGACGTGTGCTCTTCCGATCTGGGCTCGAGCGGCAGCTATTAATAGTACT	
	D711_R2	CAAGCAGAAGACGGCATACGAGATGCGCGAGAGTGACTGGAGTTCAGACGTGTGCTCTTCCGATCTGGGCTCGAGCGGCAGCTATTAATAGTACT	
	D712_R2	CAAGCAGAAGACGGCATACGAGATCTATCGCTGTGACTGGAGTTCAGACGTGTGCTCTTCCGATCTGGGCTCGAGCGGCAGCTATTAATAGTACT	
	D713_R2	CAAGCAGAAGACGGCATACGAGATGATTATTCGTGACTGGAGTTCAGACGTGTGCTCTTCCGATCTGGGCTCGAGCGGCAGCTATTAATAGTACT	

Underlined sequences represent barcode sequences.

[a] When a C-T bond was present at the terminus, oxygen atoms of the phosphate group of the oligonucleotide with normal phosphodiester bonds were exchanged for sulfate atoms to produce

[b] Primers were modified by the addition of a phosphate group at the 5' terminal.

Supplemental Table 2. Primer combinations for library construction.

Retrotransposon	Cultivar name	P5 primer	P7 primer
Tcs1	‘Miyagawa-wase’ (Satsuma mandarin)	D501_R1_Tcs1	D701_R2_1
	‘Duncan’grapefruit (Grapefruit)	D501_R1_Tcs1	D702_R2_1
	‘Trovita’orange (Sweet orange)	D501_R1_Tcs1	D703_R2_1
	‘Lisbon’lemon （Lemon）	D501_R1_Tcs1	D704_R2_1
	‘Shiranuhi’[Dekopon[a]]	D501_R1_Tcs1	D705_R2_1
	Iyo (Iyo)	D501_R1_Tcs1	D706_R2_1
	‘Kawanonatsudaidai’(Natsudaidai)	D501_R1_Tcs1	D707_R2_1
	Hassaku (Hassaku)	D501_R1_Tcs1	D708_R2_1
	‘Ohta ponkan’(Ponkan)	D501_R1_Tcs1	D709_R2_1
	‘Rinoka’	D501_R1_Tcs1	D710_R2_1
	‘Mihaya’	D501_R1_Tcs1	D711_R2_1
	‘Asumi’	D501_R1_Tcs1	D712_R2_1
	‘Asuki’	D501_R1_Tcs1	D713_R2_1
	‘Reikou’	D502_R1_Tcs1	D701_R2_1
	‘Tsunokagayaki’	D502_R1_Tcs1	D702_R2_1
	‘Seinannohikari’	D502_R1_Tcs1	D703_R2_1
	‘Tsunonozomi’	D502_R1_Tcs1	D704_R2_1
	‘Haruhi’	D502_R1_Tcs1	D705_R2_1
	‘Kiyomi’	D502_R1_Tcs1	D706_R2_1
	‘Setoka’	D502_R1_Tcs1	D707_R2_1
	‘Harumi’	D502_R1_Tcs1	D708_R2_1
	‘Harehime’	D502_R1_Tcs1	D709_R2_1
	‘Kanpei’	D502_R1_Tcs1	D712_R2_1
	‘Ehimekashidai28go’[Benimadonna[b]]	D502_R1_Tcs1	D710_R2_1
	‘Himekoharu’	D502_R1_Tcs1	D713_R2_1
	‘Ehimekashidai48go’[Beniprincess[c]]	D502_R1_Tcs1	D711_R2_1
Tcs2	‘Miyagawa-wase’ (Satsuma mandarin)	D504_R1_Tcs2	D701_R2
	‘Duncan’grapefruit (Grapefruit)	D504_R1_Tcs2	D702_R2
	‘Trovita’orange (Sweet orange)	D504_R1_Tcs2	D703_R2
	‘Lisbon’lemon （Lemon）	D504_R1_Tcs2	D704_R2
	‘Shiranuhi’[Dekopon[a]]	D504_R1_Tcs2	D705_R2
	Iyo (Iyo)	D504_R1_Tcs2	D706_R2
	‘Kawanonatsudaidai’(Natsudaidai)	D504_R1_Tcs2	D707_R2
	Hassaku (Hassaku)	D504_R1_Tcs2	D708_R2
	‘Ohta ponkan’(Ponkan)	D504_R1_Tcs2	D709_R2
	‘Rinoka’	D504_R1_Tcs2	D710_R2
	‘Mihaya’	D504_R1_Tcs2	D711_R2
	‘Asumi’	D504_R1_Tcs2	D712_R2
	‘Asuki’	D504_R1_Tcs2	D713_R2
	‘Reikou’	D506_R1_Tcs2	D701_R2
	‘Tsunokagayaki’	D506_R1_Tcs2	D702_R2
	‘Seinannohikari’	D506_R1_Tcs2	D703_R2
	‘Tsunonozomi’	D506_R1_Tcs2	D704_R2
	‘Haruhi’	D506_R1_Tcs2	D705_R2
	‘Kiyomi’	D506_R1_Tcs2	D706_R2
	‘Setoka’	D506_R1_Tcs2	D707_R2
	‘Harumi’	D506_R1_Tcs2	D708_R2
	‘Harehime’	D506_R1_Tcs2	D709_R2
	‘Kanpei’	D506_R1_Tcs2	D712_R2
	‘Ehimekashidai28go’[Benimadonna[b]]	D506_R1_Tcs2	D710_R2
	‘Himekoharu’	D506_R1_Tcs2	D713_R2
	‘Ehimekashidai48go’[Beniprincess[c]]	D506_R1_Tcs2	D711_R2
	‘Miyagawa-wase’ (Satsuma mandarin)	D507_R1_CIRE1	D701_R2_1
	‘Duncan’grapefruit (Grapefruit)	D507_R1_CIRE1	D702_R2_1
	‘Trovita’orange (Sweet orange)	D507_R1_CIRE1	D703_R2_1
	‘Lisbon’lemon （Lemon）	D507_R1_CIRE1	D704_R2_1

CIRE1	‘Shiranuhi’[Dekopon[a]]	D507_R1_CIRE1	D705_R2_1
	Iyo (Iyo)	D507_R1_CIRE1	D706_R2_1
	‘Kawanonatsudaidai’(Natsudaidai)	D507_R1_CIRE1	D707_R2_1
	Hassaku (Hassaku)	D507_R1_CIRE1	D708_R2_1
	‘Ohta ponkan’(Ponkan)	D507_R1_CIRE1	D709_R2_1
	‘Rinoka’	D507_R1_CIRE1	D710_R2_1
	‘Mihaya’	D507_R1_CIRE1	D711_R2_1
	‘Asumi’	D507_R1_CIRE1	D712_R2_1
	‘Asuki’	D507_R1_CIRE1	D713_R2_1
	‘Reikou’	D508_R1_CIRE1	D701_R2_1
	‘Tsunokagayaki’	D508_R1_CIRE1	D702_R2_1
	‘Seinannohikari’	D508_R1_CIRE1	D703_R2_1
	‘Tsunonozomi’	D508_R1_CIRE1	D704_R2_1
	‘Haruhi’	D508_R1_CIRE1	D705_R2_1
	‘Kiyomi’	D508_R1_CIRE1	D706_R2_1
	‘Setoka’	D508_R1_CIRE1	D707_R2_1
	‘Harumi’	D508_R1_CIRE1	D708_R2_1
	‘Harehime’	D508_R1_CIRE1	D709_R2_1
	‘Kanpei’	D508_R1_CIRE1	D712_R2_1
	‘Ehimekashidai28go’[Benimadonna[b]]	D508_R1_CIRE1	D710_R2_1
	‘Himekoharu’	D508_R1_CIRE1	D713_R2_1
	‘Ehimekashidai48go’[Beniprincess[c]]	D508_R1_CIRE1	D711_R2_1

[a] The registered trademark of the Federation of Kumamoto Prefectural Fruit Agriculture Cooperatives. [b] The registered trademark of National Federation of Agricultural Cooperative Associations. [c] The registered trademark of Ehime Prefectural Headquarters.

Supplemental Table 3. The number of reads in each cultivar.

Cultivar name	Read number		
	CIRE1	Tcs1	Tcs2
'Miyagawa-wase' (Satsuma mandarin)	6,110,395	2,033,783	3,876,773
'Duncan'grapefruit (Grapefruit)	5,255,083	2,054,636	5,429,866
'Trovita'orange (Sweet orange)	4,990,607	1,746,645	6,203,174
'Lisbon'lemon （Lemon）	4,250,186	1,630,221	4,391,528
'Shiranuhi'[Dekopon[a]]	6,058,505	3,542,786	4,450,630
Iyo (Iyo)	5,185,126	2,010,512	5,705,834
'Kawanonatsudaidai'(Natsudaidai)	4,929,611	1,778,564	4,695,032
Hassaku (Hassaku)	5,185,126	1,716,664	4,287,162
'Ohta ponkan'(Ponkan)	4,689,849	1,680,252	5,157,582
'Rinoka'	5,134,713	2,066,167	5,296,155
'Mihaya'	3,744,651	1,529,440	3,933,764
'Asumi'	5,155,417	2,002,069	3,671,674
'Asuki'	5,262,607	2,676,899	4,156,641
'Reikou'	5,903,308	5,003,387	6,256,825
'Tsunokagayaki'	4,920,011	4,598,344	5,873,374
'Seinannohikari'	4,709,725	3,781,638	4,785,331
'Tsunonozomi'	4,418,552	4,107,151	4,841,698
'Haruhi'	5,992,754	4,445,674	5,563,458
'Kiyomi'	3,804,695	3,365,772	5,801,947
'Setoka'	4,890,606	4,391,803	6,372,525
'Harumi'	4,193,520	3,642,748	4,399,339
'Harehime'	3,792,280	3,639,063	4,581,608
'Kanpei'	3,654,639	3,006,330	6,129,082
'Ehimekashidai28go'[Benimadonna[b]]	2,597,900	1,570,058	6,797,994
'Himekoharu'	4,084,263	3,864,985	5,860,120
'Ehimekashidai48go'[Beniprincess[c]]	3,754,603	3,760,552	6,338,944
Total	122,668,732	75,646,143	134,858,060
Minimum	2,597,900	1,529,440	3,671,674
Average	4,718,028	2,909,467	5,186,848
Maximum	6,110,395	5,003,387	6,797,994

[a] The registered trademark of the Federation of Kumamoto Prefectural Fruit Agriculture Cooperatives. [b] The registered trademark of National Federation of Agricultural Cooperative Associations. cThe registered trademark of Ehime Prefectural Headquarters.

Supplemental Table 4. Summary of reads in the data analysis for CIRE1.

Analysis	No. of reads	Ratio (%)	No. of collapsed reads (more than 10)	No. of clusters
Raw data	122,668,732	100.00	-	
Adaptor removal and QV (>=30) filtering	121,453,172	99.01	88,450	
Trimming to specific length (50bp) and QV (>=30) filtering	74,639,965	60.85	8,092	
Outlier filtering	57,420,094	46.81	3,976	
BLAT clustering				93

Supplemental Table 5. Summary of reads in the data analysis for Tcs1.

Analysis	No. of reads	Ratio (%)	No. of collapsed reads (more than 10)	No. of clusters
Raw data	75,646,143	100.00	-	
Adaptor removal and QV (>=30) filtering	75,594,128	99.93	77,074	
Trimming to specific length (50bp) and QV (>=30) filtering	45,764,459	60.50	9,587	
Outlier filtering	37,259,503	49.26	5,471	
BLAT clustering				276

Supplemental Table 6. Summary of reads in the data analysis for Tcs2.

Analysis	No. of reads	Ratio (%)	No. of collapsed reads (more than 10)	No. of clusters
Raw data	134,858,060	100.00	-	
Adaptor removal and QV (>=30) filtering	134,747,907	99.92	155,906	
Trimming to specific length (50bp) and QV (>=30) filtering	55,977,139	41.51	9,265	
Outlier filtering	33,251,578	24.66	4,393	
BLAT clustering				174
